# An ultra-compact rejection filter based on spoof surface plasmon polaritons

**DOI:** 10.1038/s41598-017-11332-8

**Published:** 2017-09-05

**Authors:** Shumin Zhao, Hao Chi Zhang, Jiahao Zhao, Wen Xuan Tang

**Affiliations:** 1 Beijing Innovation Center for Future Chip, State Key Laboratory of Precision Measurement Technology and Instruments, Department of Precision Instruments, Tsinghua University, Beijing, 100084 P.R. China; 2 Luoyang Optoelectro Technology Development Center, Luoyang, 471009 P.R. China; 3 School of Information Science and Engineering, Southeast University, Nanjing, 210096 P.R. China

## Abstract

In this paper, we propose a scheme to construct a new type of ultra-compact rejection filter by loading split-ring resonators (SRRs) on the transmission line of spoof surface plasmon polaritons (SPPs). From the dispersion analysis of the spoof SPP transmission line with and without the SRR loading, we clearly reveal the mechanism of the rejection characteristic for this compact filter. Meanwhile, we fabricate two spoof SPPs waveguides loaded with different amounts of metamaterials particles, and experimentally test them using an Agilent Vector Network Analyzer (VNA) and a homemade near-field scanning system. Both the simulated and measured results agree well with our theoretical analysis and demonstrate the excellent filtering characteristics of our design. The isolation of both filters can be less than −20 dB, and even reach −40 dB at rejection frequencies. The proposed rejection and stop-band filters show important potentials to develop integrated plasmonic functional devices and circuits at microwave and terahertz frequencies.

## Introduction

Due to the shortage of spectrum resource, the filter plays a very important role in modern wireless communication system^1^. In real application, a broadband system need to reject some specifically reserved spectrum, for example, the Wi-Fi channel frequencies. Hence, the rejection filter is widely used in wireless communication system^2–4^. However, in traditional technology, the rejection filter is an independent device, which leads to extra loss. In order to overcome this problem, we proposed a method to control the electromagnetic (EM) field in sub-wavelength scale through the interaction between spoof surface plasmon polaritons (SPPs) and metamaterials (MTMs) particles, and consequently control the frequency spectrum of the whole circuit or system.

In fact, the concept of SPPs is brought from the optical community. The SPPs wave is a special kind of surface wave at optical frequencies, which is formed by the interaction between the free electron in metal and the EM field around the metal^5^. From the view of EM theory, SPPs exist in the interface between two media with opposite dielectric constants^6^. The SPPs wave propagates along the interface and decays exponentially in the direction perpendicular to the interface, leading to the features of field confinement and enhancement. Hence, SPPs have spurred great interest in physics during the past decades and inspired many potential applications in optoelectronics, biochemical detection and optical circuitsminiaturization^7–12^.

However, the excellent characteristics of SPPs are still in great demand in low-frequency circuits because the optical design cannot be directly used in low frequency bands (i.e. far infrared, terahertz, and microwave) because metals act as perfectly electric conductor (PEC) other than plasma with negative permittivity. In order to overcome this difficulty, researchers have proposed spoof (or designer) SPPs to support the surface wave mode behaving alike the natural SPPs mode^8–13^. In the early stage, considering the convenience to acquire analytic solutions, the structures of spoof SPPs are in a thick manner that is not applicable in planar circuits. Accordingly, a series of planar SPPs structures have been proposed and proved compatible with traditional planar circuit technology^14–23^. In addition, the SPP circuits possess unique merits from the basic physical view, such as reducing the dielectric loss^24^, breaking the signal integrity challenges^25^, reducing the size of shielding box^26^ and reducing the bending loss. Up to date, a series of passive and active devices based on planar spoof SPP have been proposed in order to construct spoof SPP based circuits and systems with high performance.

However, we have noticed that, as one of the most important components in communication circuits, the spoof-SPP-based filter usually has a larger size when compared with the traditional microstrip filter. Therefore, here, we proposed an ultra-compact spoof SPP based rejection filter, which is composed of three parts: (1) the conversion part; (2) the spoof SPPs waveguide and (3) the split-ring resonators (SRRs) embed in the spoof SPPs waveguide. Through the analysis of the dispersion curve, we explained the theory of controlling the frequency spectrum, and gave a scheme to design a new kind of rejection filter. Meanwhile, we presented numerical and experimental results to demonstrate the good filtering performance. The designing method, as well as the features of filtering SPP waves, can be significant for the following-up development of plasmonic integrated circuits and systems.

## Results

**Figure 1:**
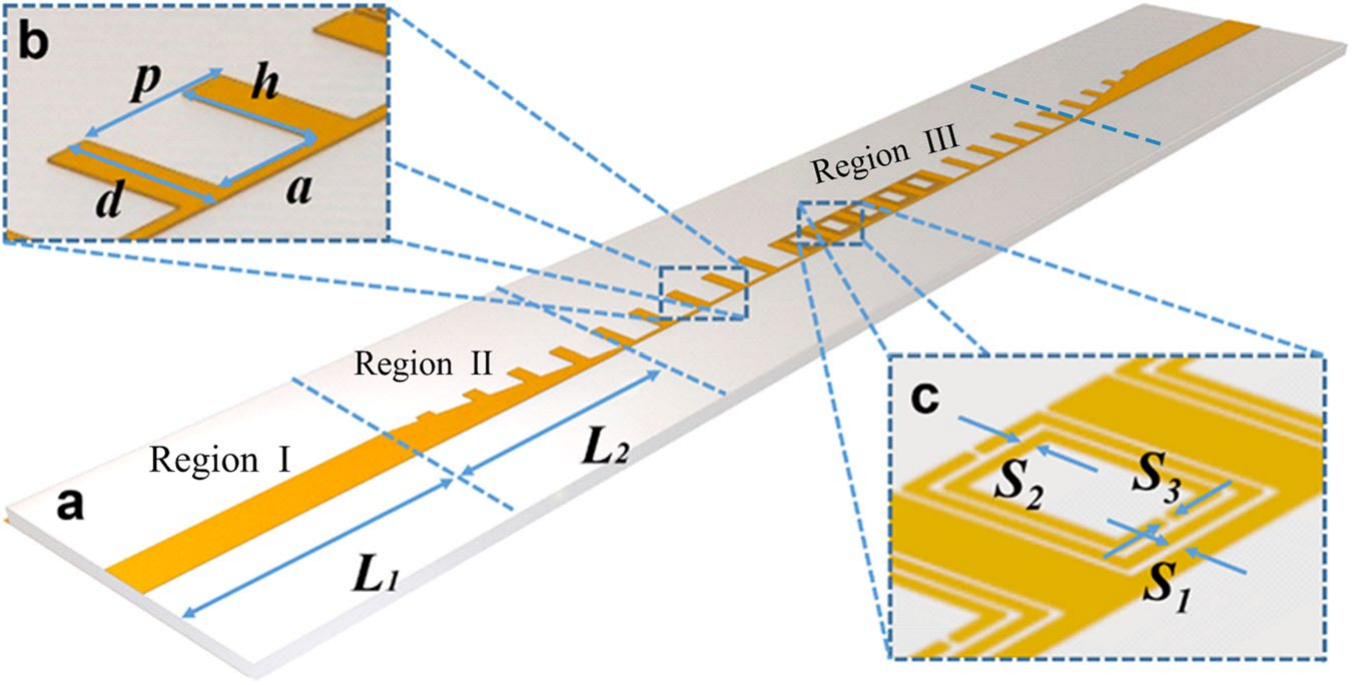
Schematic diagrams of spoof SPPs rejection filter. (**a**) Schematic diagram of the spoof SPPs rejection filter, which can be divided into three regions: (1) the microstrip with length *L*_1_, (2) the conversion section with length *L*_2_, (3) the SPP transmission line with rejection units. (**b**) Schematic diagram of the spoof SPP unit cell, which is described by the period *p*, the width of the groove *a*, the depth of the groove *h*, the strip width *d*, the metal thickness *t*, and the substrate thickness *t*_*d*_. (**c**) Schematic diagram of the SRRs, which is described by the gap between the SPPs and the SRR structure *S*_1_, the width of metallic strip of SRRs *S*_2_, and the width of spit in the SRRs *S*_3_.

The proposed band-rejection filter is illustrated in Fig. 1, containing the spoof SPPs waveguide and specifically located SRRs units. The designed filtering circuit is printed on a thin and flexible dielectric film, F4B, whose relative permittivity is 2.65 and thickness is 0.8 mm. The annealed copper (with electric conductivity σ = 5.8 e + 007 S/m) is used as the metal layer and the ground with the thickness of 0.018 mm. For facilitating the experiment with standard SMA connectors, we design two microstrip sections at the input and output respectively, as is illustrated in Fig. 1. However, as is known, the microstrip line supports a quasi-transverse electromagnetic (TEM) mode whilst the spoof SPPs structure supports a TM mode. Hence, to achieve the impedance and momentum matching, a conversion section that contains a series of SPPs units with gradient grooves^26^ is introduced into our design. The depth of the gradient grooves is linearly varied with a slope of *h*/*L*_2_, where *h* = 2 mm refers to the depth of the groove in a common spoof SPP unit and *L*_2_ = 12.15 mm, as is noted in Fig. 1.

For the main transmission section, we adopt a kind of common spoof SPPs structure that consists of corrugated metallic strip on the top of the dielectric substrate and a completely metallic ground on the bottom side, as shown in Fig. 1(b). The period is *p* = 5.5 mm, the width of strip is d = 2.2 mm, and the width and depth of groove are *a* = 1.8 mm and *h* = 2 mm, respectively. In this design, the main transmission section includes 15 units and the total length of this filter is 86.5 mm, which is remarkably smaller than that of the previous spoof SPPs rejection filter (342.5 mm)^27^. The SRRs is one of the most widely used metamaterial particles to provide sub-wavelength scaled magnetic resonance. An SRR unit is usually constructed with two concentric rings with opposite splits. As sketched in Fig. 1(c), the SRRs are integrated into the groove of the SPPs units. The gap between two rings, the width of the ring and the width of the split are chosen as *S*_1_ = 0.1 mm, *S*_2_ = 0.1 mm and *S*_3_ = 0.1 mm, respectively.

**Figure 2:**
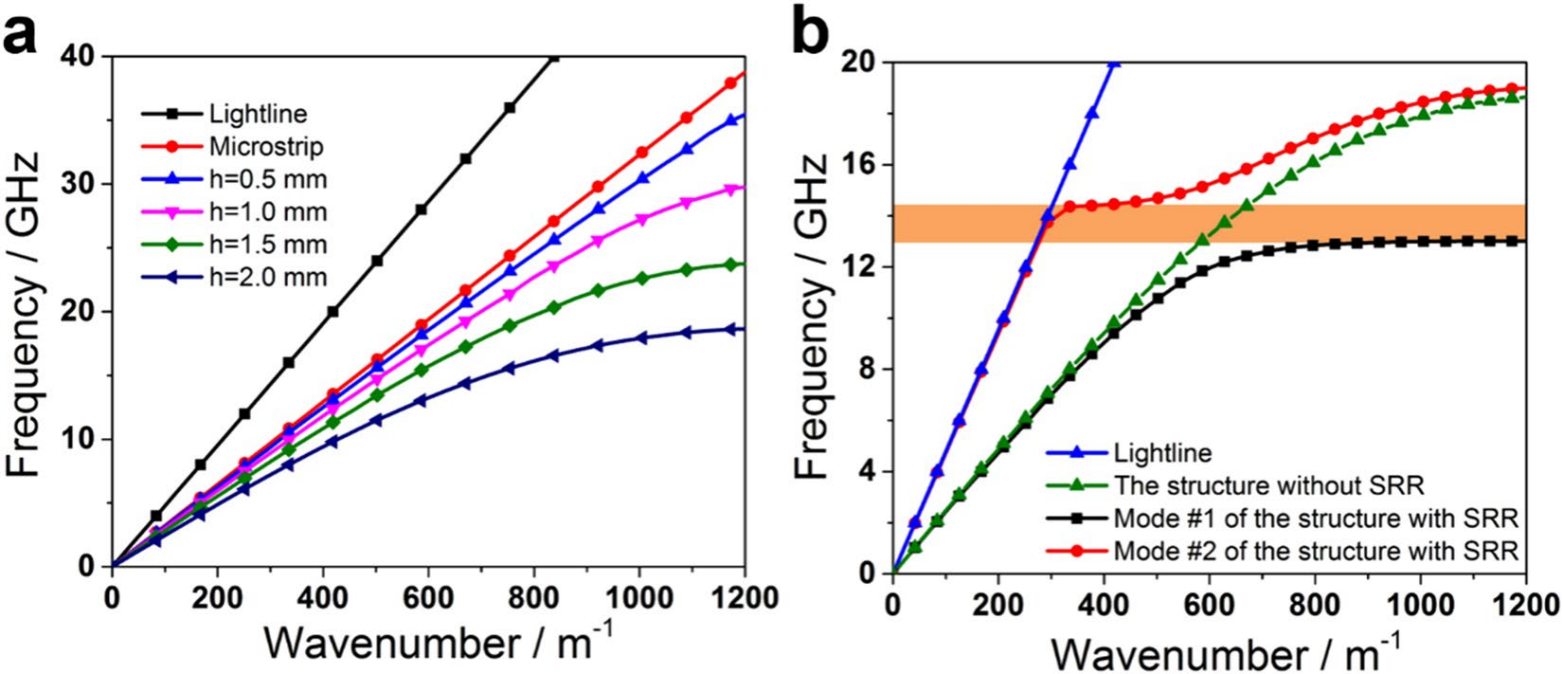
Dispersion curves of the spoof SPPs TLs. (**a**) Dispersion curves of the spoof SPPs TLs with different groove depths *h*, in which strip width *d* = 2.2 mm, groove width *a* = 1.8 mm, and period *p* = 2.5 mm. (**b**) Dispersion curves of different spoof SPPs TLs, in which strip width *d* = 2.2 mm, groove width *a* = 1.8 mm, period *p* = 2.5 mm, and groove depth *h* = 2 mm.

In order to analyze the propagating characteristic of this device, dispersion curves of the spoof SPPs structures are investigated using Eigen-mode solver in the commercial software of CST Microwave Studio. In the simulation course, periodic boundary conditions are set to a SPPs unit along the x-axis whilst electric walls are set along both y- and z-axes 50 mm away from the SPPs unit. The loss of materials (including metal and dielectric) is ignored to simplify the Eigen mode simulation and reduce the complexity of calculation. According to the previous research^28, 29^, ignoring the metal loss would produce a tiny blue shift in the spectrum but have negligible influence on the shape of the dispersion curve. Figure 2(a) depicts the dispersion curves of SPPs transmission lines (TLs) with different groove depths. From this figure, we can clearly observe that all of the dispersion curves exhibit SPP-like behaviors, which gradually deviate from the light-line and then asymptotically approach different cutoff frequencies. More importantly, the performance of the slow wave becomes more and more striking as the groove depth *d* increases from 0.5 mm to 2.0 mm, which allows easy tuning of wave momentum and hence guarantees smooth conversion between the spoof SPP waves and the conventional guided waves. Furthermore, we investigated the change of dispersion curve when the SRRs are integrated into the groove of spoof SPPs, as shown in Fig. 2(b). This figure shows the dispersion curve of the SPPs TL integrated with SRRs, and a forbidden band is found between the pass-bands of mode #1 and mode #2, implying that the EM waves cannot propagate in this frequency band. In fact, an SPPs TL with *n* SRRs can be regarded as a long SPPs waveguide with wave number *k*_1_(*ω*) inserted by a short SPP-SRRs waveguide section with wave number *k*_2_(*ω*). Hence, the reflection coefficient *R*(*ω*) is then given as follow according to ref. 30.

1 $$R(\omega )={[1+{[\frac{2{k}_{1}(\omega ){k}_{2}(\omega )}{[{k}_{1}^{2}(\omega )-{k}_{2}^{2}(\omega )]\sin ({k}_{2}(\omega )np)}]}^{2}]}^{-1}.$$

And, if we ignore the loss of propagation, the transmission coefficient *T*(*ω*) can also be written as

2 $$T(\omega )=1-R(\omega ).$$

Hence, according to Eqs (1) and (2), for an incident wave at low frequency, the difference between *k*_1_(*ω*) and *k*_2_(*ω*) is very small and the reflection coefficient is very low as well. However, when the frequency increases, the difference between *k*_1_(*ω*) and *k*_2_(*ω*) gradually expands and the reflection coefficient gradually increases accordingly. When the frequency of incident wave falls into the forbidden band, the total reflection happens and *R*(*ω*) approaches 1. If the frequency continues to increase, the reflection will decrease until the frequency goes close to the cut-off frequency of the second mode of the spoof SPPs TL.

**Figure 3:**
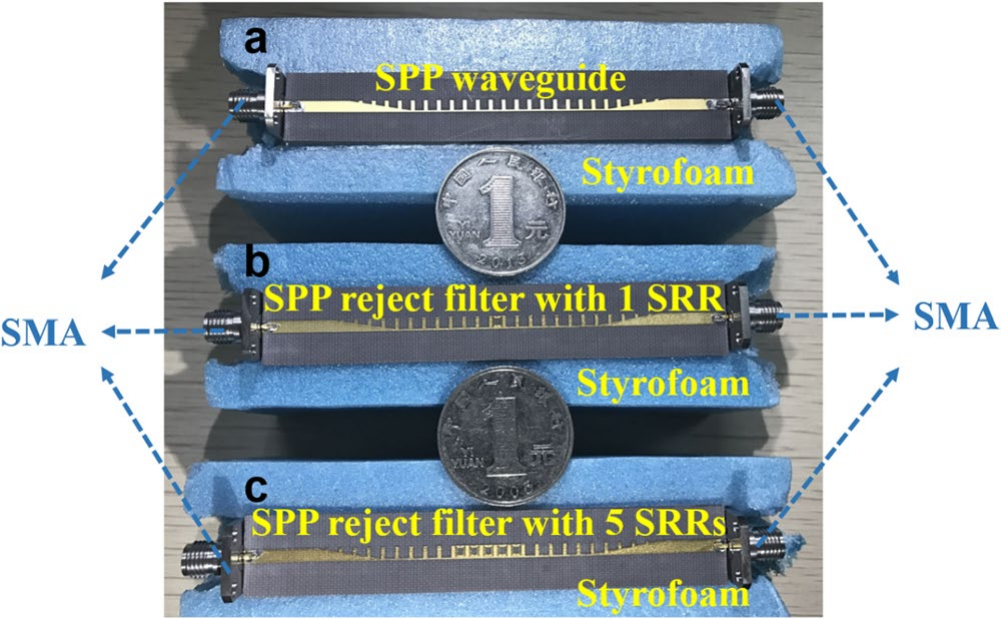
The photographs of the fabricated (**a**) SPPs TL, (**b**) SPPs rejection filter with one SRR unit and (**c**) SPPs rejection filter with five SRRs units. The geometric parameters are shown in Table 1.

**Figure 4:**
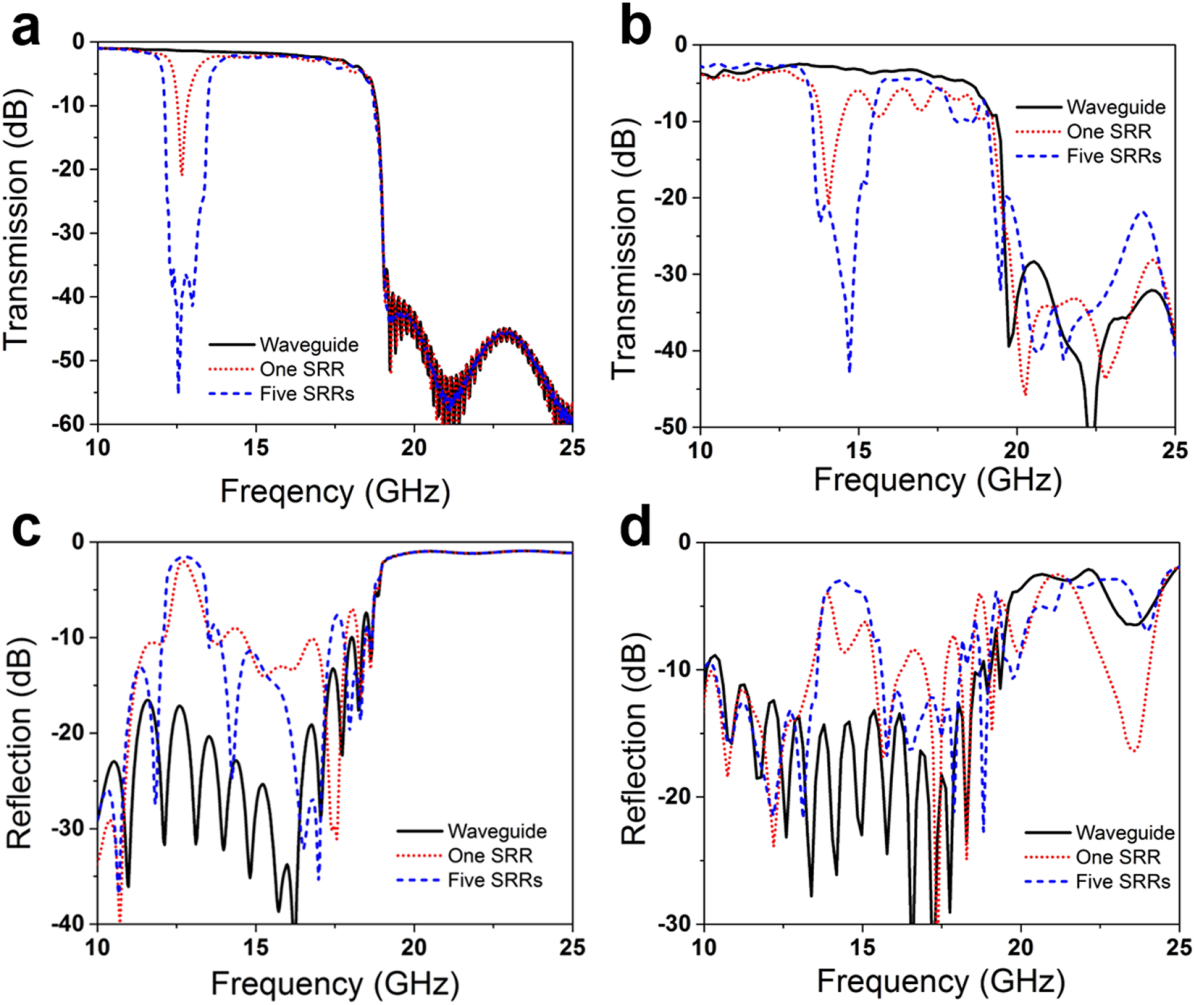
Simulated (**a**,**c**) and measured (**b**,**d**) transmission and reflection coefficients of the spoof SPPs TL and two kinds of spoof SPPs rejection filters. The geometric parameters are shown in Table 1.

**Figure 5:**
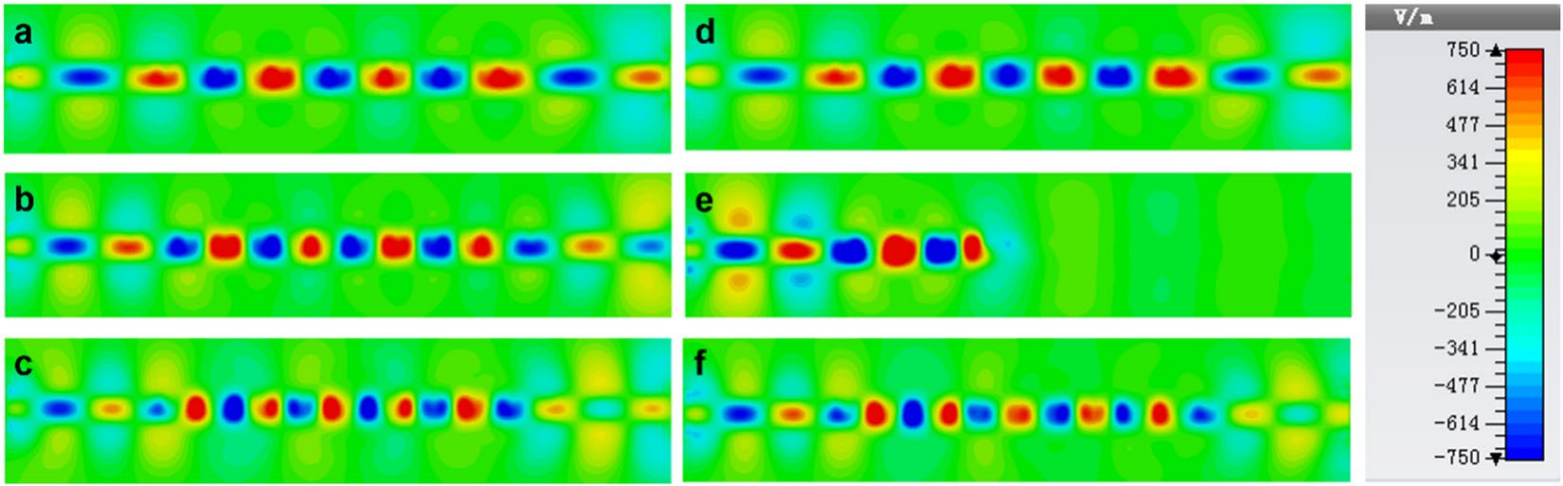
The simulated near-electric-field distributions of the spoof SPPs TL and spoof SPPs rejection filter with 5 SRRs in a plane 1 mm above the dielectric substrate at different frequencies. (**a**–**c**) The simulated results at (**a**) 10 GHz (in the pass band), (**b**) 12.7 GHz (in the forbidden band), and (**c**) 15 GHz (in the pass band) for the spoof SPPs TL. (**d**–**f**) The simulated results at (**d**) 10 GHz (in the pass band), (**e**) 12.7 GHz (in the forbidden band), and (**f)** 15 GHz (in the pass band) for the spoof SPPs rejection filter with five SRRs.

**Figure 6:**
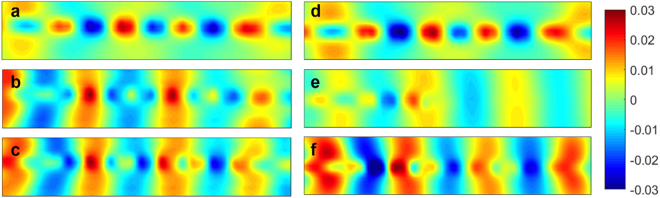
The measured near-electric-field distributions of the spoof SPPs TL and spoof SPPs rejection filter with 5 SRRs in a plane 1 mm above the dielectric substrate at different frequencies. (**a**–**c**) The measured results at (**a**) 10 GHz (in the pass band), (**b**) 13.7 GHz (in the forbidden band), and (**c**) 15 GHz (in the pass band) for the spoof SPPs TL. (**d**–**f**) The measured results at (**d**) 10 GHz (in the pass band), (**e**) 13.7 GHz (in the forbidden band), and (**f**) 15 GHz (in the pass band) for the spoof SPPs rejection filter with five SRRs.

Based on the dispersion analysis, the SRR-integrated spoof SPP structure can behave as a rejection filter. In order to verify this idea, two kinds of spoof SPPs rejection filter with one and five SRR structures respectively, as well as a simple spoof SPPs TL for comparison, are fabricated using the printed circuit board (PCB) technology. The three prototypes (as depicted in Fig. 3) have the same geometrical parameters that are listed in Table 1. Meanwhile, we connect the input and output ports to the measurement system through SMA connectors and coaxial cables with an insertion loss about 1 dB. Numerical simulation is carried out using time domain solver in the CST Microwave Studio and measurement is executed using the vector network analyzer (VNA). Simulated and measured transmission and reflection coefficients for the three samples are sketched in Fig. 4. In this figure, both the simulated and measured transmission and reflection coefficients demonstrate the rejection performance of the filters. The rejecting band (the forbidden band) locates around 12.5 GHz in simulation and around 13.5 GHz in measurement. The extra loss in measurement may be led by the inhomogeneity of the dielectric substrate and the loss of SMA connector, respectively. On the other hand, the frequency shift between the simulated and experiment result may lead by the machining error, because in our scheme, the gap between the SPPs and the SRR structure *S*_1_, the width of metallic strip of SRRs *S*_2_ are set as 0.1 mm, which is equal to machining accuracy. To be specific, for the one SRRs case, we can find that only the EM wave around the rejecting frequency will be reflected, but for the five SRRs case, the most of EM energy is reflected from 12 to 13.5 GHz for simulated (from 13 to 14.5 GHz for experiment result. Hence, adding the SRRs units is help to improve the filtering performance. Meanwhile, we also notice the stop-band beyond the cut-off frequency of the spoof SPPs. In order to visually verify the rejecting performance of the design, we show the simulated and measured near electric field distribution of the spoof SPPs TL and the rejection filter with five SRRs, as shown in Figs 5 and 6. The near electric field is plotted in a plane 1 mm above the dielectric substrate. In the pass-band at low frequencies, the EM wave propagates along both the SPPs TL and the spoof SPPs rejection filter, thanks to the small reflection between the spoof SPPs TL and the SRRs-loaded spoof SPPs TL, as shown in Fig. 5a and d. However, in the forbidden band, the EM wave cannot propagate along the SRRs-loaded spoof SPPs TL and energy is reflected, as is shown in Fig. 5e. In contrast, the EM wave can propagate along the simple spoof SPPs TL, as is sketched in Fig. 5b. When the frequency is between the forbidden frequency and the cut-off frequency of the second mode of the rejection filter, the EM wave can propagate again along the spoof SPPs rejection filter as if there are no SRRs loaded, as shown in Fig. 5f. Figure 6(a–f) depicts the corresponding results of the near-electric -field distributions measured by a home-made near-field scanning system, which have very good agreements with the simulated results. Both the simulated and measured results intuitively provide a proof to corroborate the great filtering performance for the plasmonic circuits at microwave frequencies.

**Table 1:** The geometric parameters for three samples (Unit: mm).

Parameters	Sample #1	Sample #2	Sample #3
Number of SRRs	0	1	5
Number of SPPs	15	15	15
*L*_1_	12.35	12.35	12.35
*L*_2_	12.15	12.15	12.15
*p*	2.5	2.5	2.5
*h*	2.0	2.0	2.0
*d*	2.2	2.2	2.2
*a*	1.8	1.8	1.8
*S*_1_	N.A.	0.1	0.1
*S*_2_	N.A.	0.1	0.1
*S*_3_	N.A.	0.1	0.1

**Figure 7:**
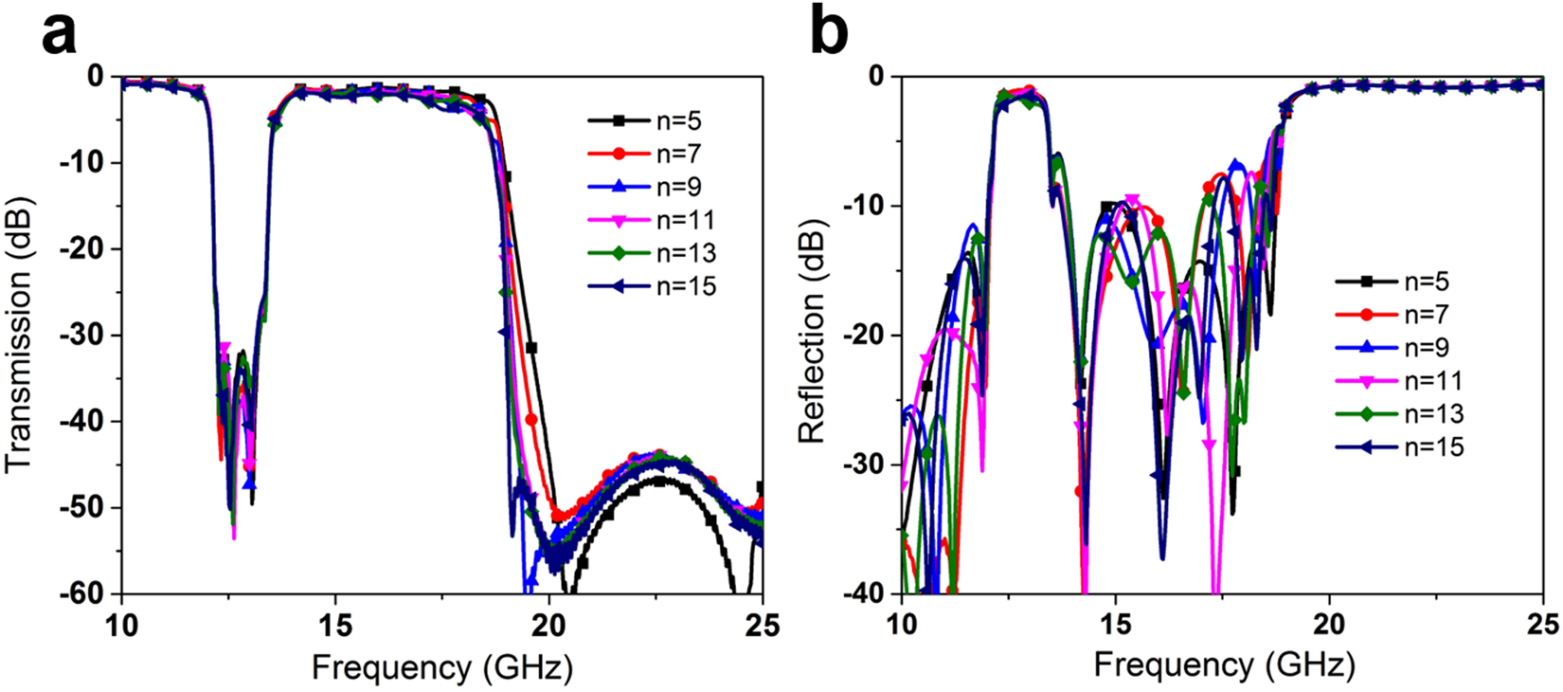
Simulated transmission (**a**) and reflection (**b**) coefficients of the spoof SPPs rejection filters with different number of SPPs unit (including SPPs unit with and without SRRs).

Last, we discuss the miniaturization possibility of this design. Because the width of the 50-Ohm microstrip, the width of our design cannot further compress, which is very difficult for original design. Hence, we only need discuss the possibility of reducing the size in the propagation direction. Based on the above analysis, five SPP-SRR units can provide an enough rejection filtering performance for almost common application. However, the filtering performance will deteriorate if we reduce the number of SPP-SRR unit. Hence, we adopt the five SPP-SRRs design in the discussion of this miniaturization. Under this condition, the possible method to reduce the size is reducing the SPPs unit without SRRs. Here, we give the simulation result of simulated transmission and reflection coefficients of the spoof SPPs rejection filters with different number of SPPs unit (including SPPs unit with and without SRRs), as shown in Fig. 7. The dispersion performance of the SPP-SRR unit also can provide the cut-off frequency for high frequency, but which is higher than the cut-off frequency of SPP units, as shown in Fig. 2b. Hence, the cut-off frequency of the rejection filter will tiny blue shift, as shown in Fig. 7a. If we recognized the tiny blue shift is acceptable, the miniaturization whole length of our design is 41.5 mm, which is less than double of wavelength at center of rejected band.

## Conclusion

By mean of integrating SRRs into the SPP TL, we have presented two kinds of compact band-rejection filters to develop highly efficient and controllable rejection-band plasmonic circuits. In virtue of the highly confined electromagnetic waves of SPPs, the proposed filters have excellent transmission efficiency and remarkable band-stop feature. Meanwhile, based on the dispersion analysis, there exists forbidden bands where signal cannot propagate. Based on this principle, we have designed rejection filters using spoof SPP TLs integrated with SRRs. Prototype filters have been manufactured for experimentally verifying the marvelous capability. Both simulation and measurement results have demonstrated the highly efficient filtering performance. This work provides potentials to promote the development of plasmonic functional devices and integrated circuits in both microwave and terahertz frequencies.
